# Increased glycolipid storage produced by the inheritance of a complex intronic haplotype in the α-galactosidase A (*GLA*) gene

**DOI:** 10.1186/s12863-015-0267-z

**Published:** 2015-09-03

**Authors:** Javier Gervas-Arruga, Jorge J. Cebolla, Pilar Irun, Javier Perez-Lopez, Luis Plaza, Jose C. Roche, Jose L. Capablo, Jose C. Rodriguez-Rey, Miguel Pocovi, Pilar Giraldo

**Affiliations:** Centro de Investigación Biomédica en Red de Enfermedades Raras (CIBERER), Zaragoza, Spain; Translational Research Unit, Instituto de Investigación Sanitaria Aragón (IIS Aragón), Miguel Servet University Hospital, Zaragoza, Spain; Instituto de Investigación Sanitaria Aragón (IIS Aragón), Zaragoza, Spain; Biochemistry and Molecular and Cellular Biology Department, Universidad de Zaragoza, Zaragoza, Spain; Molecular Biology Department, Cantabria University and IFIMAV, Santander, Spain; Anatomic Pathology Department, Miguel Servet University Hospital, Zaragoza, Spain; Neurology Department, Miguel Servet University Hospital, Zaragoza, Spain

**Keywords:** *GLA*, Fabry disease, Haplotypes, Galactosphingolipids, α-galactosidase A

## Abstract

**Background:**

Accumulation of galactosphingolipids is a general characteristic of Fabry disease, a lysosomal storage disorder caused by the deficient activity of α-galactosidase A encoded by the *GLA* gene. Although many polymorphic *GLA* haplotypes have been described, it is still unclear whether some of these variants are causative of disease symptoms. We report the study of an inheritance of a complex intronic haplotype (CIH) (c.-10C > T, c.369 + 990C > A, c.370-81_370-77delCAGCC, c.640-16A > G, c.1000-22C > T) within the *GLA* gene associated with Fabry-like symptoms and galactosphingolipid accumulation.

We analysed α-Gal A activity in plasma, leukocytes and skin fibroblasts in patients, and measured accumulation of galactosphingolipids by enzymatic methods and immunofluorescence techniques. Additionally, we evaluated *GLA* expression using quantitative PCR, EMSA, and cDNA cloning.

**Results:**

CIH carriers had an altered *GLA* expression pattern, although most of the carriers had high residual enzyme activity in plasma, leukocytes and in skin fibroblasts. Nonetheless, CIH carriers had significant galactosphingolipid accumulation in fibroblasts in comparison with controls, and also glycolipid deposits in renal tubules and glomeruli. EMSA assays indicated that the c.-10C > T variant in the promoter affected a nuclear protein binding site.

**Conclusions:**

Thus, inheritance of the CIH caused an mRNA deregulation altering the *GLA* expression pattern, producing a tissue glycolipid storage.

**Electronic supplementary material:**

The online version of this article (doi:10.1186/s12863-015-0267-z) contains supplementary material, which is available to authorized users.

## Background

α-galactosidase A (α-Gal A, EC3.2.1.22) is a lysosomal enzyme that hydrolyses the terminal α-galactosyl moieties from glycolipids. α-Gal A is encoded by the *GLA* gene and mutations in this gene causes deficiency or absence of the enzyme, resulting in Fabry disease (FD) (OMIM 301500), an X-linked inherited lysosomal storage disorder. This disease leads to accumulation of globotriaosylcermide (Gb_3_), globotriaosylsphingosine (lyso-Gb_3_), galabiosylceramide (Ga_2_) and neutral glycosphingolipids in lysosomes of several tissues, mainly in the endothelium of the vascular tree [1]. Depending on the *GLA* mutation and when manifestations initially occur, FD is classified as late-onset or classic phenotype. In males with no, or reduced, α-Gal A enzyme activity, clinical manifestations of the classic form include acroparesthesias, angiokeratomas, hypohidrosis, corneal and lenticular opacities, cardiac dysfunction and brain and renal involvement with proteinuria [2]. Heterozygous females can either be asymptomatic, due to random X-chromosomal inactivation [3], or can develop the classic phenotype [1]. Because of the nonspecific nature of its clinical manifestations, FD often remains undiagnosed in concordance with the prevalence calculated in some studies [4, 5]; however, early treatment is essential to avoid significant disease progression [6, 7].

Over 700 *GLA* mutations, including missense and nonsense mutations, rearrangements, and splicing defects, have been identified as causing FD [8] (http://www.hgmd.cf.ac.uk/ac/index.php). *GLA* [ENSG:00000102393] has seven splice variants (http://www.ensembl.org) and aberrant splicing accounts for ~5 % of mutations in FD (reported to the Human Gene Mutation Database; http://www.hgmd.cf.ac.uk), but information on pre-mRNA splicing is only available for a limited number of patients. Exonic mutations may also alter splicing, but are not easily recognized [9]. Many polymorphic *GLA* variants have been described, but it is unclear if haplotypes formed by combinations of these variants correlate with FD. A complex intronic haplotype (CIH) within the *GLA* gene (c.-10C > T, c.640-16A > G, c.1000-22C > T) is associated with early occurrence of small-fibre neuropathy [10]. A screening study found a second CIH (c.-10C > T, c.370-81_370-77delCAGCC, c.640-16A > G, c.1000-22C > T) in 8.9 % of subjects with FD symptoms [11]; associated with hypertrophic heart disease [12] and, additionally, an analysis of the male control population of a suspected FD female index case permitted the identification of seven different *GLA* haplotypes [13]. Finally, a recent study described a large family with four male/female carriers of a CIH who developed a classical phenotype with a mild renal and neurological involvement [14]. Some of these intronic variants have been reported as polymorphic variants in general population [15–17].

We performed a functional characterization of a large family with a CIH in the *GLA* gene and investigated the molecular pathological mechanisms associated with different FD-related clinical manifestations. We demonstrate that the inheritance of this CIH causes *GLA* gene deregulation and glycolipid storage.

## Methods

### Patients

We studied a family with different FD-related clinical manifestations. This family carried a CIH (c.-10C > T [rs2071225], c.369 + 990C > A [rs1023431], c.370-81_370-77delCAGCC [rs5903184], c.640-16A > G [rs2071397], c.1000-22C > T [rs2071228]). The family consisted of 15 individuals (6 heterozygous and 4 hemizygous for CIH) aged between 7 and 72 years. Written informed consent was obtained from all patients including those from the parents on the behalf of the minors involved in our study. The study was approved by the Ethics Committee of Aragon (CEICA) and was conducted in accordance with the Declaration of Helsinki of 1975, as revised in 2008.

### Reagents

The recombinant enzyme used was agalsidase alfa (REPLAGAL) from Shire pharmaceuticals. As a source of enzyme, we used the residual amounts of the reconstituted recombinant enzyme prepared for the treatment of FD patients. TNFα was purchased from Miltenyi Biotec. The pharmacological chaperone, DGJ (1-deoxygalactonojirimycin hydrochloride), was purchased from Santa Cruz Biotechnology. The anti-human CD77 (Gb_3_) primary rat monoclonal antibody used for immunofluorescence analysis was purchased from Biorbyt; the anti-human CD17 (lactosylceramide) mouse monoclonal antibody was from Santa Cruz Biotechnology and the anti-human LAMP1 rabbit monoclonal antibody was from Sigma-Aldrich. Secondary conjugated antibodies were Alexa Fluor 546 goat anti-rabbit IgG, Alexa Fluor 488 goat anti-rat IgG and Alexa Fluor 488 goat anti-mouse IgG, all from Life Technologies.

### α-Gal A activity assay

The activity of α-Gal A was determined in plasma, lysed leukocytes and lysed fibroblasts (in triplicate) with a fluorometric assay using the artificial substrate 4-methylumbelliferyl (MU)-α-D-galactopyranoside (Sigma-Aldrich); for the assay of cell extracts, N-acetyl-D-galactosamine (Sigma-Aldrich) was used to inhibit α-galactosidase B (α-Gal B) [18]. The fluorescence of released 4-MU was measured in two replicates at an excitation wavelength of 366 nm and emission wavelength of 445 nm in a fluorometer (Perkin Elmer LS-45).

### Fibroblast culture

Human skin fibroblasts were cultured in 175 cm^2^ flasks in Dulbecco’s modified Eagle’s medium (DMEM) supplemented with GlutaMAX, 10 % heat-inactivated fetal bovine serum (FBS), 0.5 % β-amphotericin (250 μg/mL) and the antibiotics streptomycin (100 mg/L) and penicillin (100 U/L). Cells were maintained at 37 °C in a 5 % CO_2_ atmosphere and the medium was replenished every 72 h. After reaching confluence, cells were kept quiescent for 5 days, washed with PBS and harvested by trypsin (0.05 % trypsin/EDTA for 5 min). All reagents were provided by Gibco-Invitrogen. Cell suspensions were pelleted by centrifugation at 290×g for 5 min. After removing the supernatant, cells were washed twice with PBS and stored at −20 °C for further analysis.

### Cell culture model of lysosomal storage dysregulation

Fibroblasts were cultured in DMEM with 1 % inactivated FBS in 6-well culture plates (160, 000 cells/ 9.5 cm^2^). Twenty-four hours before experimentation, cells were activated with 0.1 nM TNFα for 16 h. After activation, the medium was renewed and, where indicated, cells were additionally incubated with DGJ (500 μM) for 24 h to inhibit endogenous α-galactosidase activity [19] or with agalsidase alfa, by adding 3 ml of the enzyme solution (1.32 μg of enzyme per ml; final activity 3.8 μmol MU mg^−1^.h^−1^) [20], to analyze galactolipid degradation as an endogenous control. Cell cultures were incubated for different periods of time (0, 16 and 24 h) and at least two 9.5 cm^2^ wells were analyzed for each treatment combination.

### Quantification of galactosphingolipids

Determination of galactosphingolipids using galactose oxidase was carried out in cell pellets from lysed leukocytes and fibroblasts previously suspended in 1 % sodium taurocholate (Sigma-Aldrich) and disrupted by combination of ultrasound short pulse (VibraCell) and timing-out on ice. Samples were kept on ice during the procedure. The lysate was centrifuged at 112×g for 10 min at 4 °C to eliminate cell debris and 50 μl of supernatant was used for quantification. Measurement of galactosphingolipids was performed using the Amplex Red Galactose/Galactose Oxidase Assay Kit (Invitrogen).

### Immunofluorescence analysis

Immunocytochemistry was performed to examine the distribution of CD77, CD17 and LAMP1. Fibroblasts grown on coverslips were fixed with 4 % paraformaldehyde for CD77/LAMP1 analysis and with methanol for CD17/LAMP1 localization, permeabilized with 0.1 % saponin and blocked with 0.1 % saponin 5 % BSA diluted in PBS. Cells were incubated with primary antibodies (CD77 diluted to 50 μg/ml, CD17 diluted to 2 μg/ml and LAMP1 diluted to 0.2 μg/ml) overnight at 4 °C and then conjugated secondary antibodies diluted to 0.1 μg/ml were added and were incubated for 20 min at 25 °C. Coverslips were mounted on slides and covered with 5 μl of DAPI-Mowiol medium (Life Technologies/Calbiochem). All samples were examined with an Olympus FluoView FV10i confocal microscope under identical conditions. We used 405 nm, 473 nm and 635 nm excitation lasers, which were switched on separately to reduce crosstalk of the two fluorochromes. A threshold was applied to the images to exclude ∼ 99 % of the signal found in control images. Pixel and cell surface fluorescence quantification was done using FV10-ASW 3.1 software from Olympus.

### Molecular *GLA* analysis

Genomic DNA was isolated from whole blood by standard procedures. The entire *GLA* gene (g.101409127-g.101397726) including promoter, exons and introns, was amplified by PCR using the primers described in Additional file 1. Amplicons were purified and sequenced in an automated DNA sequencer (3500XL genetic analyser, Applied Byosistems). Sequences were compared with the genomic *GLA* reference sequence [ENSG:00000102393].

### Multiplex Ligand Probe Amplification (MLPA) *GLA* analysis

Patients F1.1, F1.4 and F1.10 were analysed by MLPA technique in order to find any pathological rearrangement in *GLA* gene. The details of the MLPA probe target regions and test methodology are described by MRC Holland (http://www.mlpa.com). SALSA MLPA P159 GLA probemix, version A2 (MRC Holland, Netherlands) was used and data were normalized using 3 healthy controls matched by age and sex.

### RNA isolation, cDNA synthesis, small RNA cloning, and quantitative real-time PCR

Total RNA was isolated from patients and control peripheral blood samples using the PAXgene Blood RNA Kit (PreAnalytiX). RNA integrity was assessed by agarose gel electrophoresis and concentration was determined in a NanoVue 4282 V1.7.3 Spectrophotometer (GE Healthcare). Total RNA (1 μg) was reverse transcribed in triplicate 20 μL reactions using the SuperScript II reverse transcriptase Kit (200 units) and Oligo(dT)_12–18_ (500 μg/mL) (Invitrogen). The RT-PCR profile was: 10 min at 42 °C, 45 min at 60 °C, and 5 min at 95 °C.

To identify splicing variants, the entire *GLA* mRNA was amplified by PCR using 1 μL cDNA and the primers described in Additional file 1. The amplification conditions were: 2 min at 94 °C followed by 40 cycles of 20 s at 94 °C, 30 s at 57 °C, and 30 s at 72 °C, and a final extension of 72 °C for 4 min. Amplicons were diluted and nested PCR was performed to amplify the *GLA* mRNA in two parts. Both amplicons were purified with ExoSap-IT (GE Healthcare) and sequenced.

A fragment of the 3′ region of intron 6 (g.101398149-g.101398099) was amplified from cDNA of CIH carriers by nested PCR using miScript PCR system and miScript Primer Assays (Qiagen) trying to identify small RNA fragments. The product was cloned into the pGEM-T Easy Vector (Promega) and purified with the PureLink Quick Plasmid Miniprep Kit (Invitrogen). Inserts were amplified by PCR with T7 and SP6 universal primers, and products were sequenced in forward and reverse directions.

Quantification of *GLA* mRNA was performed in triplicate samples from each patient using a quantitative real-time PCR (qPCR) method based on TaqMan technology (Applied Biosystems). Probe and primers for wild-type *GLA* (ENST00000218516) and *GLA*-M (cloned fragment) are listed in Additional file 1. qPCR was performed with the ABI Prism 7000 Sequence Detector (PE Applied Biosystems) using 20 ng of cDNA in a reaction mixture containing 10 μl Universal Master Mix without amperase, 300 nmol of each primer and 200 nmol of probe (Additional file 1). Conditions for qPCR were: 95 °C for 10 min followed by 40 cycles of 95 °C for 15 s and 60 °C for 1 min. We used efficiency-corrected gene expression measurements [21].

### Electrophoretic mobility shift assay (EMSA)

IRDye680-labelled double-stranded oligonucleotides with the sequence GCTGTCCGGT[C/T]ACCGTGACAA were purchased from LICOR Biosciences (Lincoln, NE, USA). The preparation of nuclear extracts and the electrophoresis procedures have been described previously [22]. EMSA results were analysed and quantified using the Odyssey Infrared Imaging System (LICOR Biosciences). For competition assays, an excess of unlabelled T-allele oligonucleotide was added to the mix prior to the addition of the labelled oligonucleotides. The inverse of band intensity was plotted against the excess of unlabelled oligonucleotide and the slope of the resulting straight line indicated the affinity of each allele for the proteins in the nuclear extract.

### Splice-site score (SSS)

Splice mutations were analysed using the SSPNN program (http://www.fruitfly.org/seq_tools/splice.html) and a splice-site score (SSS) was obtained.

### Statistical analysis

Statistical analysis was carried out using the SPSS software package (IBM). Normality of the distribution of variables was analysed by the Kolmogorov-Smirnov test, and mean comparison by the parametric *t*-test and one way ANOVA. Differences with *p* ≤ 0.05 were considered statistically significant.

## Results

### Patients

**Fig. 1:**
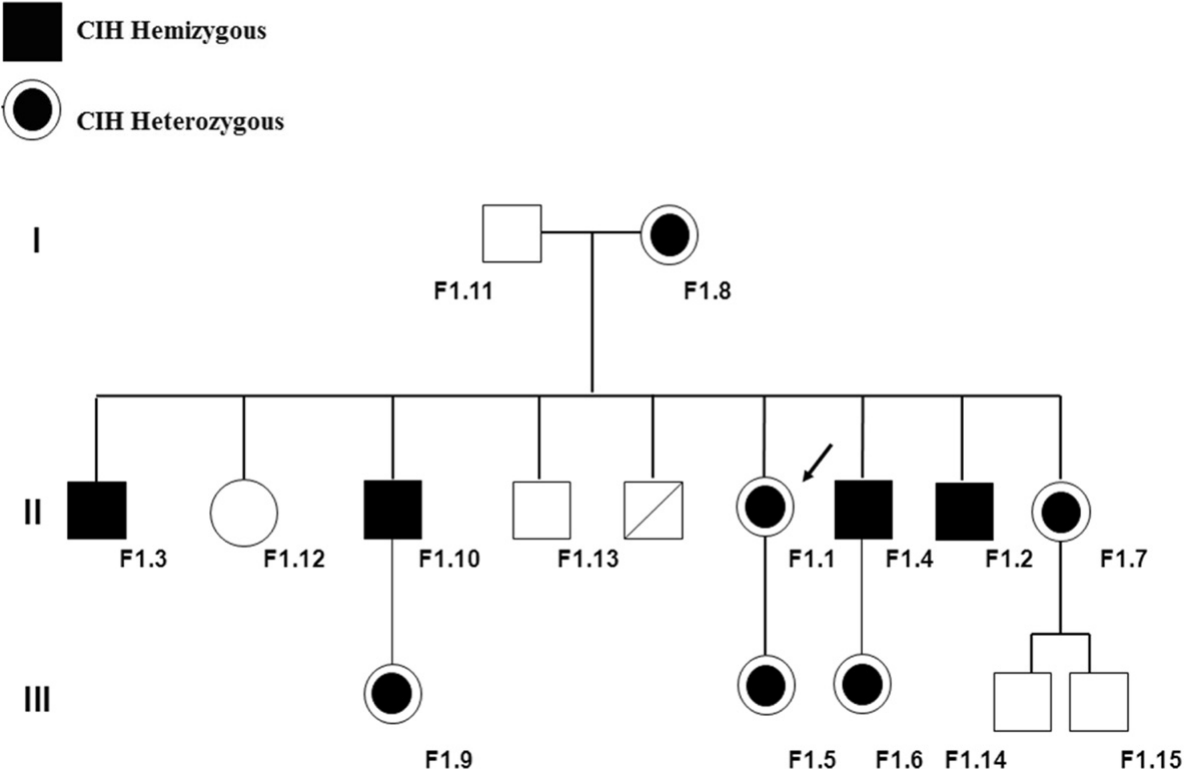
Pedigree of the family and complex intronic haplotype (c.-10C > T, c.369 + 990C > A, c.370-81_370-77delCAGCC, c.640-16A > G, c.1000-22C > T) carriers. Index case is indicated with an arrow

The family pedigree is shown in Fig. 1. The index case (F1.1) was a 43-year-old female, with clinical records of acroparesthesias from adolescence, hypohidrosis, and reiterated episodes of abdominal pain with alternating phases of diarrhoea with constipation. At 40 years of age was referring several episodes of pain chest that requiring emergency care. At 41 years of age she was hospitalized by a new episode of chest pain and was diagnosed with anteroseptal myocardial infarction, requiring the implantation of three stents. The evaluation of hearth function by MRI showed residual fibrous scar by myocardial infarction in the territory of the anterior and apex segments. LV slightly dilated with decreased LVEF (48.4 %) and generalized hypokinesia. Valvular function with MI moderate to severe and moderate AI. The cardiologic diagnosis suspicion was valvular and ischemic hearth variant secondary to Fabry disease. Complete haematological, neurological, and ophthalmological examinations were performed, and renal function was measured and determined to be normal. It discard associate factors of hypercoagulability status. Peripheral nervous conduction was evaluated by quantitative sensory test (QST) [23], which revealed abnormalities in the small fiber conduction compared with normal population. Physical examination indicated that the patient had absence of hemangiokeratomas, nodes or visceral enlargement, and no cardiovascular risks such as high plasmatic cholesterol, low c-HDL levels or hypertension. Genetic analysis revealed a CIH in the *GLA* gene: IVSO-10C > T (c.-10C > T) within the promoter region, intron 2 IVS2 + 990C > A (c.369 + 990 C > A); IVS2-76_80del 5 (c.370-77_81 del CAGCC), intron 4 IVS4-16A > G (c.640-16A > G) and intron 6 IVS6-22C > T (c.1000-22C > T). The same haplotype was identified in the patient’s mother (F1.8) and daughter (F1.5), in four of her brothers (F1.2, F1.3, F1.4, and F1.10) and one of her two sisters (F1.7), as well as in the daughters (F1.6, F1.9) of two of the affected brothers. MLPA assays did not reveal any copy number differences in patients F1.1, F1.4 and F1.10.

At age 52 years, the first brother (F1.3) was referred for examination to exclude malignant monoclonal gammopathy IgG-kappa. At age 40 years, he underwent vertebral fixation surgery because of severe back pain. He reported acroparesthesia, heat intolerance, and hypohidrosis since childhood, as well as shortness of breath and inability to perform any physical activity. This patient was hemizygous for the CIH.

The second brother (F1.10), hemizygous for the CIH, was 49 years old. His main symptoms were related to left ventricular hypertrophy and valvular aortic stenosis associated with severe acral pain that demanded continuous intake of analgesic drugs.

The third brother (F1.13) did not accept to be a part of the study and the fourth brother was deceased.

**Fig. 2:**
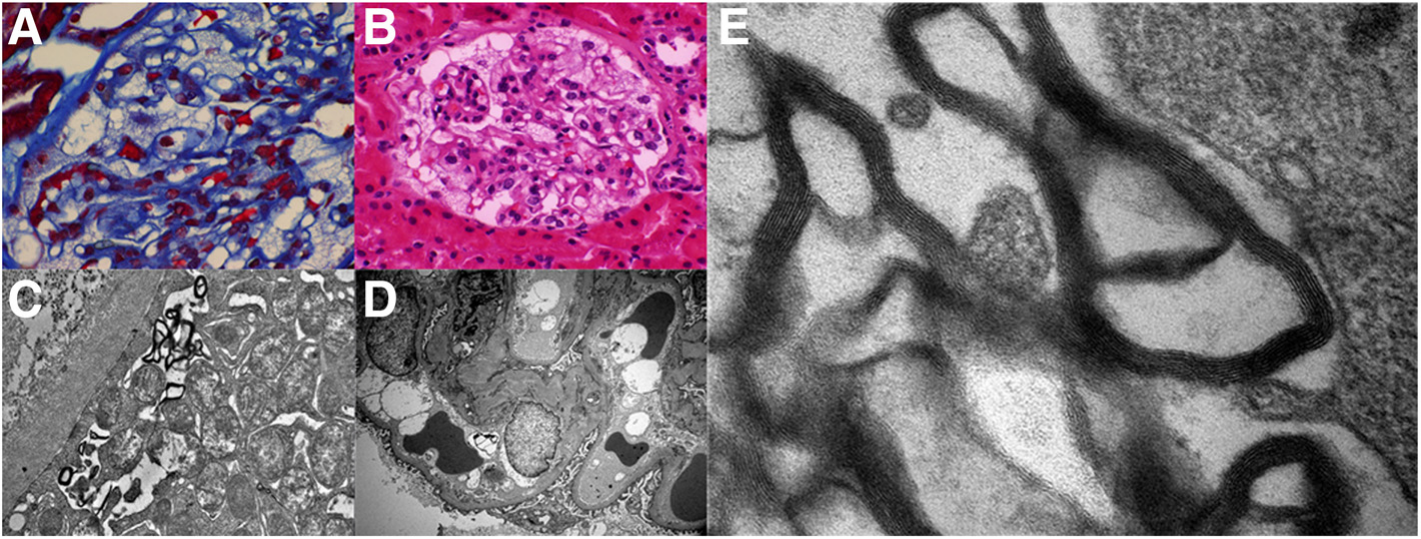
Cytoplasmic vacuolation observed in a podocyte by light microscopy with **a** Masson’s trichrome and **b** Haematoxylin and eosin staining. **c** Myelin-like structures in a podocyte, with concentric lamellated ultra-structural appearance. (Electron Microscopy). **d** Focal areas of podocyte effacement; amorphous myelin-like structures are visible in glomerular parietal epithelial cells and in endothelial cells (Electron Microscopy). **e** Myelin-like structures parallel with zebra-like body appearance (Electron Microscopy)

The fifth brother (F1.4) was referred for hearing loss and mild acroparesthesia at 41 years of age. Renal involvement was characterised by microalbuminuria (34 mg/24 h) and glycolipid deposits in renal tubules and glomeruli (Fig. 2). It is important to note that this patient did not use chloroquine or amiodarone-like drugs. His 7-year-old daughter (F1.6) had microalbuminuria (29 mg/24 h), and was heterozygous for the CIH.

The sixth brother (F1.2) was 39 years old, hemizygous for the CIH, and was referred for acroparesthesias, bilateral hearing loss, heat intolerance and hypohidrosis, but no cardiac abnormalities were observed.

One sister (F1.7) was 37 years old and a carrier of the CIH. She had a previous history of tachyarrhythmias, and since childhood she had acroparesthesias, hypohidrosis and heat intolerance. Her mother (F1.8) presented no clinical manifestations.

### α-Gal A activity in plasma, leukocytes and fibroblasts

**Fig. 3:**
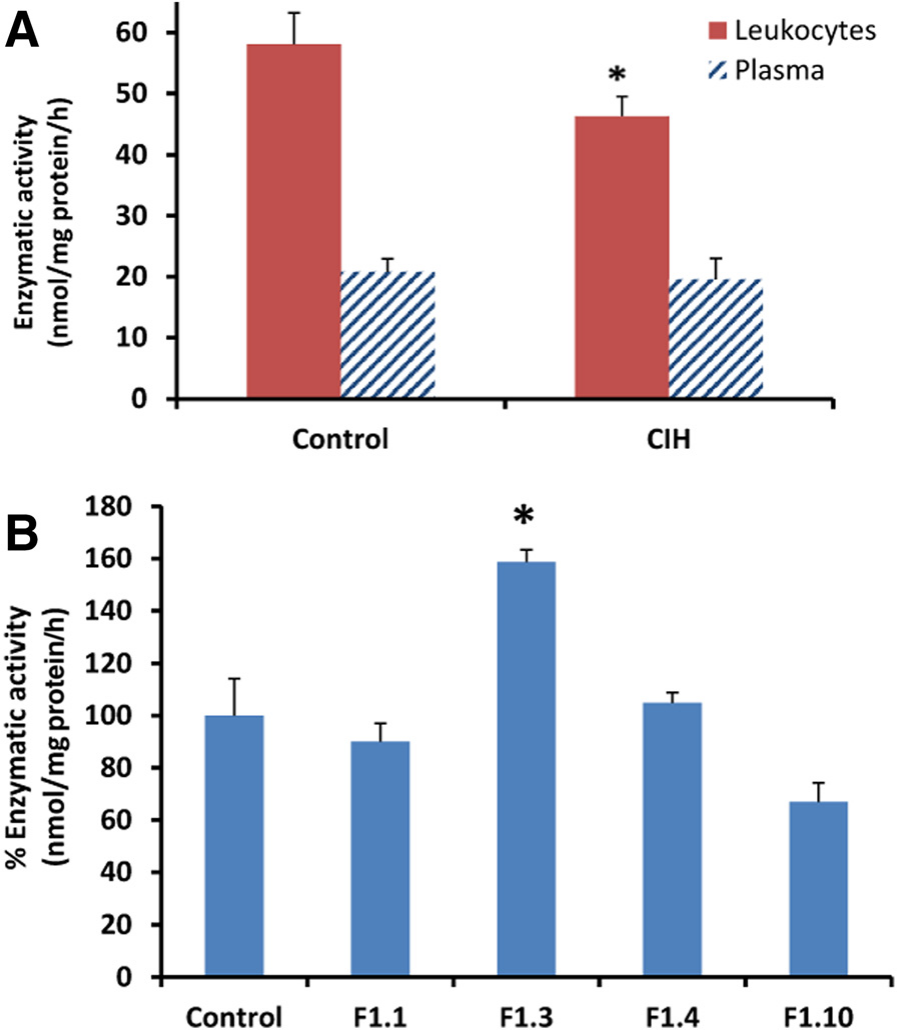
α-Gal enzyme activity. **a** Enzymatic activity in lysed leukocytes (nmol/mg protein/hour) and plasma (nmol/mL/hour), represented as mean ± SEM. **b** Enzymatic activity in lysed fibroblasts (nmol/mg protein/hour) represented as mean ± SEM, *n* = 3 *p ≤ 0.05

Enzymatic activity was measured in plasma, lysed leukocytes and fibroblasts from CIH carriers and normal healthy controls. The individual values from leukocytes and plasma are presented in Table 1. The mean value of lysed leukocyte activity in the control group (*n* = 27) was (mean ± SD) 58.1 ± 26.6 nmol/mg protein/h. Significantly lower levels were found in the CIH group (*n* = 8), 46.25 ± 9.66 nmol/mg protein/h (Fig. 3a; *p* ≤ 0.05). The mean value of plasma activity in the control group (*n* = 33) was (mean ± SD) 20.8 ± 12.5 nmol/mL/h, whereas the mean value for the CIH group (*n* = 9) was 19.5 ± 10.3 nmol/mL/h. There was no significant difference between the plasma activity in control and CIH group (Fig. 3a). Enzymatic activity was also measured in cultured skin fibroblasts from controls and from patients F1.1, F1.3, F1.4 and F1.10. The activity measurements in the patients’ fibroblasts were not significantly different to controls, although there was a trend for reduction in patient F1.10 and fibroblasts from patient F1.3 had significantly higher levels of α-Gal A (Fig. 3b).

**Table 1:** Clinical manifestations; α-Gal A activity in plasma and leukocytes, galactosphignolipid concentrations and *GLA* mRNA expression

ID	SEX	Age	Variants	Clinical Manifestations	galactosphingolipids	galactosphingolipids	α-Gal A	α-Gal A	mRNA	mRNA
			CIH		Leukocytes	Fibroblasts	Leukocytes	Plasma	GLA	GLA-M
					(nmol/mg protein)	(nmol/mg protein)	(nmol/mg protein/h)	(nmol/mL/h)	RQ	RQ
F1.1	f	43	Het	Ischemic heart disease, Gastro- intestinal, Fine fiber alterations	0.62	0.83	63	23	0.48^*^	0.85
F1.2	m	39	Hemi	Acroparesthesias, Heat intolerance, Hypohidrosis, Hearing loss	0.13	N/A	57	19	0.41	5.47
F1.3	m	53	Hemi	Acroparesthesias, Heat intolerance, Hypohidrosis	0.11	0.80	38	14	0.36^*^	0.84
F1.4	m	41	Hemi	Hearing loss, Microalbuminuria, Renal deposits	0.11	1.12	37	15	0.88	2.56
F1.5	f	20	Het	N/A	0.13	N/A	42	37	N/A	N/A
F1.6	f	7	Het	Microalbuminuria	N/A	N/A	N/A	9	N/A	N/A
F1.7	f	37	Het	Acropaesthesias, Microalbumminuria, Hypohidrosis, Tachyarrhythmias	0.06	N/A	43	15	1.06	1.51
F1.8	f	72	Het	N/A	0.27	N/A	51	35	1.12	0.90
F1.10	m	49	Hemi	Left Ventricular Hypertrophy	1.93	0.93	39	9	0.02^**^	117.76^**^

CIH = c.-10C > T [rs2071225], c.369 + 990C > A [rs1023431], c.370-81_370-77delCAGCC [rs5903184], c.640-16A > G [rs2071397], c.1000-22C > T [rs2071228]. The mean ± SD normal concentration of leukocytes galactosphingolipids is (*n* = 17) 0.33 ± 0.3 (nmol/mg protein). The mean ± SD normal concentration of fibroblasts galactosphingolipids is (*n* = 5) 0.63 ± 0.12 (nmol/mg protein). The normal mean ± SD of α-Gal A leukocyte activity in our assay is (*n* = 27) 58.1 ± 26.6 (nmol/mg protein/hour) and normal mean ± SD of α-Gal A plasma activity is (*n* = 33) 20.8 ± 22.03 (nmol/mL/ hour). ^*^ = *p* < 0.05, ^**^ = *p* < 0.001. N/A = Not applicable

### *In vitro* model of lysosomal storage disease

**Fig. 4:**
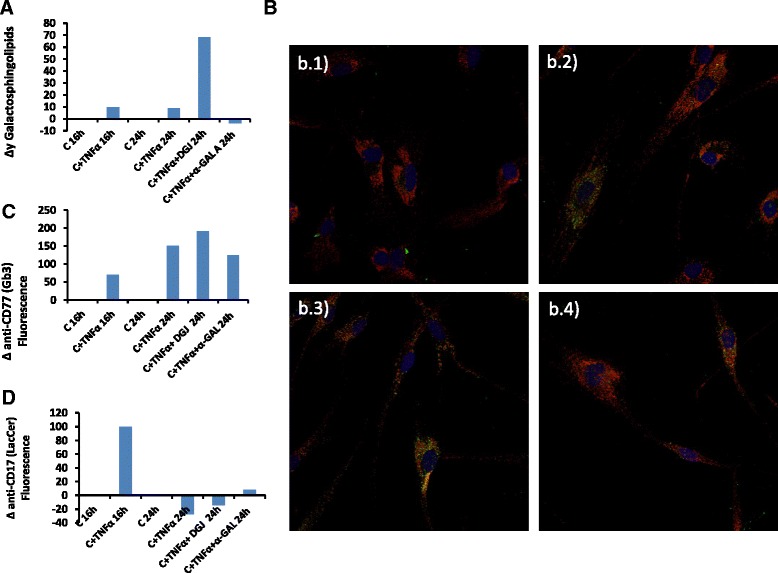
Model of FD substrate accumulation *in vitro*. **a** Galactosphingolipid variation rate in wild-type fibroblasts under different conditions. Galactosphingolipids concentration was measured as described (*n* = 3) and changes in galactosphingolipids levels are represented. **b** Representative images of *in vitro* FD model. Control fibroblasts were stained with CD77 (Gb_3_), green and LAMP1, red. CD77 and LAMP1 merged appear as orange/yellow. Nuclei were stained with DAPI, blue. *b.1*) Control 24 h; *b.2*) Control + TNFα 24 h; *b.3*) Control + TNFα 24 h + DGJ; b*.4*) Control + TNFα 24 h + agalsidase alfa. **c** Quantification of CD77/LAMP1 (orange/yellow) fluorescence rate (*n* = 3). **d** Quantification of CD17/LAMP1 (orange/yellow) fluorescence rate (*n* = 3)

In order to accurately determine galactosphingolipid levels, we first used wild-type fibroblasts to establish a model of lysosomal storage dysregulation. Fibroblasts were treated or not with TNFα (16 h) to activate a proinflammatory response, followed by a medium change without TNFα, and culturing was then continued for a further 8 h. Accumulation of galactosphingolipids was measured by galactose oxidase assay at 16 h and 24 h has described. TNFα treatment resulted in an increase in galactosphingolipids in wild-type fibroblasts compared with control samples (without TNFα treatment) (Fig. 4a). Notably, this increase was accentuated by co-treatment of fibroblasts with TNFα and DGJ, used at a concentration which inhibits endogenous α-Gal A activity [19] (Fig. 4a). Moreover, addition of recombinant agalsidase alfa significantly decreased the level of endogenous galactosphingolipids in TNFα-treated fibroblasts with respect to control cells (Fig. 4a). To corroborate these results, immunofluorescence microscopy was performed to evaluate the presence of Gb_3_ (CD77) using an antibody to CD77 (Fig. 4b). In good agreement with the biochemical assay, immunofluorescence staining of fibroblasts exposed to TNFα demonstrated an increase in CD77, which co-localized with the lysosomal membrane marker LAMP1. Additionally, Gb_3_ signal intensity was increased upon exposure of fibroblasts to TNFα and DGJ (Fig. 4b). Superimposition of CD77/LAMP1 images revealed a significant degree of overlap (Fig. 4b). As anticipated, agalsidase alfa treatment reduced Gb_3_ accumulation as demonstrated by decreased CD77 fluorescence, without affecting LAMP1 staining (Fig. 4b). Quantification of overlapped pixels of confocal images revealed that TNFα treatment resulted in a 50 fold increase in CD77 staining in fibroblasts, which further increased to 150 fold after 24 h (Fig. 4c). Co-treatment with DGJ further increased CD77 staining and this was dramatically reduced upon exposure to agalsidase alfa (Fig. 4c). A similar analysis was performed with an antibody to lactosylceramide (CD17). α-Gal A catalyzes the hydrolysis of Gb_3_ to lactosylceramide and α-galactose. Lactosylceramide acumulation was decreased upon exposure of fibroblasts to TNFα and DGJ. Agalsidase alfa treatment increased lactosylceramide accumulation as demonstrated by increased CD17 fluorescence, corroborating the CD77 results (Fig. 4c). Collectively, these results show that galactosphingolipids can be precisely measured in cell cultures using this assay and immunocytochemistry is a useful technique to assess lipid storage.

### Quantification of galactosphingolipids in leukocytes and human fibroblasts

**Fig. 5:**
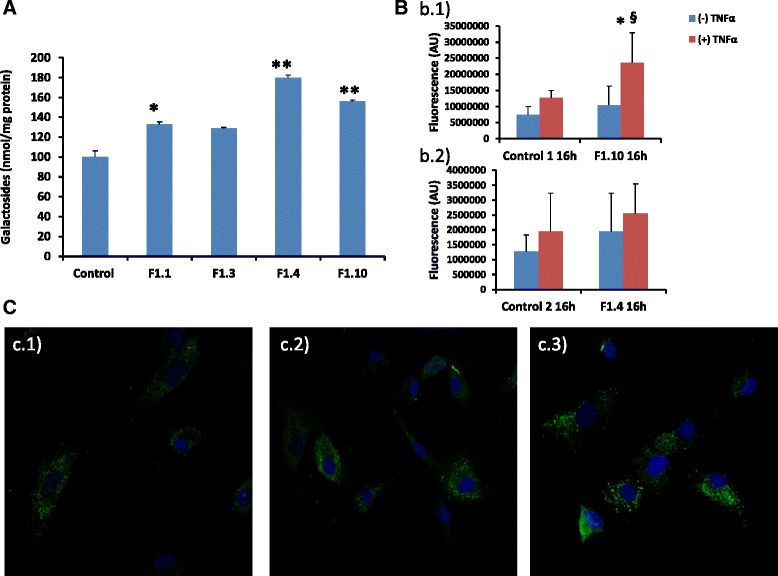
Accumulation of galactosphingolipids in fibroblast lysates. **a** Biochemical quantification after 5 days of culture represented as mean ± SEM. (*n* = 3) *p ≤ 0.05 **p ≤ 0.001. **b** Quantification of Gb_3_ (CD77) confocal images represented as mean ± SD (*n* = 3) *b.1*) patient F1.10.*p ≤ 0.05; (+)TNFα vs. (−)TNFα and ^#^p ≤ 0.05; Control vs. F1.10., *b.2*) Patient F1.4. **c** Immunocytochemistry of CD77 expression in fibroblasts from *c.1*) control, *c.2*) patient F1.4 and *c.3*) patient F1.10 after 16 h TNFα activation. Nuclei were stained with DAPI, blue

Quantification of galactosphingolipids was performed in leukocytes and fibroblasts from CIH carriers and controls. The individual values of leukocyte galactosphingolipids are presented in Table 1. The mean value for the control group (*n* = 17) was (mean ± SD) 0.33 ± 0.30 nmol/mg protein and in the CIH group (*n* = 8) 0.42 ± 0.63 nmol/mg protein. Galactosphingolipids were also measured in cultured fibroblasts from CIH patients. After 5 days of quiescence, in all cases galactosphingolipid levels in patient fibroblasts were greater than in equivalent control cell lines, which was significant for patients F1.1, F1.4 and F1.10 (Fig. 5a). Interestingly, in patients F1.3 and F1.4, leukocyte levels were lower than controls, whereas in equivalent fibroblasts the levels were higher. The origin of these differences may reflect distinct *GLA* expression states of different cells. Ferreira et al., suggest that the GLA 5’UTR polymorphysms are a possible modulators of *GLA* expression varying among different cell types [24]. Atypical FD variants are often not associated with increased lyso-Gb3 levels, although biopsies of affected organs revealed lamellar inclusion bodies characteristic for FD [25]. Consistent with the increase in galactosphingolipids, immunostaining of fibroblasts from patient F1.4 and F1.10 revealed elevated levels of Gb_3_, which were further increased after exposure to TNFα (Fig. 5b). Additionally, immunofluorescence revealed that galactolipids were mostly confined to cytosolic and membrane compartments (Fig. 5b). Quantification of CD77/LAMP1 lysosomal co-localization revealed an increase in CD77 staining in fibroblasts from patients F1.4 and F1.10, which was significant for patient F1.10 relative to control values (Fig. 5c).

### cDNA analysis

No sequence changes were discovered after sequencing nested PCR products of the two *GLA* amplicons in comparison with the reference sequence (data not shown).

### Small RNA cloning

Sequencing of the 3′ region of intron 6 insert in all CIH carriers revealed the presence of one cDNA fragment formed by a 49-bp portion of intron 6 and exon 7 cloned only in patient F1.4. The genomic coordinates of this exon 7 extension are: ChrX(GRCh38): g.101398050-g. 101397803 :-1.

### Quantitative PCR of the GLA transcripts

Relative quantification (RQ) of wild-type (wt) *GLA* and *GLA*-M mRNA (3′ region of intron 6 cloned fragment) from all carriers and controls (*n* = 8; 50 % females) was performed by real-time PCR and the *GLA*/*GLA-*M expression profiles were compared between control group and carriers. Reduced wt *GLA* expression was found in all hemizygous carriers and in the heterozygous proband F1.1, and was significant in carriers F1.3, F1.10 and F1.1 (Table 1). The relative expression of *GLA*-M was increased in carriers F1.2, F1.4 and F1.7, and significantly in patient F1.10 compared with controls (Table 1). The heterozygous group for CIH presented higher RQ values for *GLA* wt expression in comparison with the hemizygous group (0.89 vs 0.41).

### Electrophoretic mobility shift assay (EMSA)

**Fig. 6:**
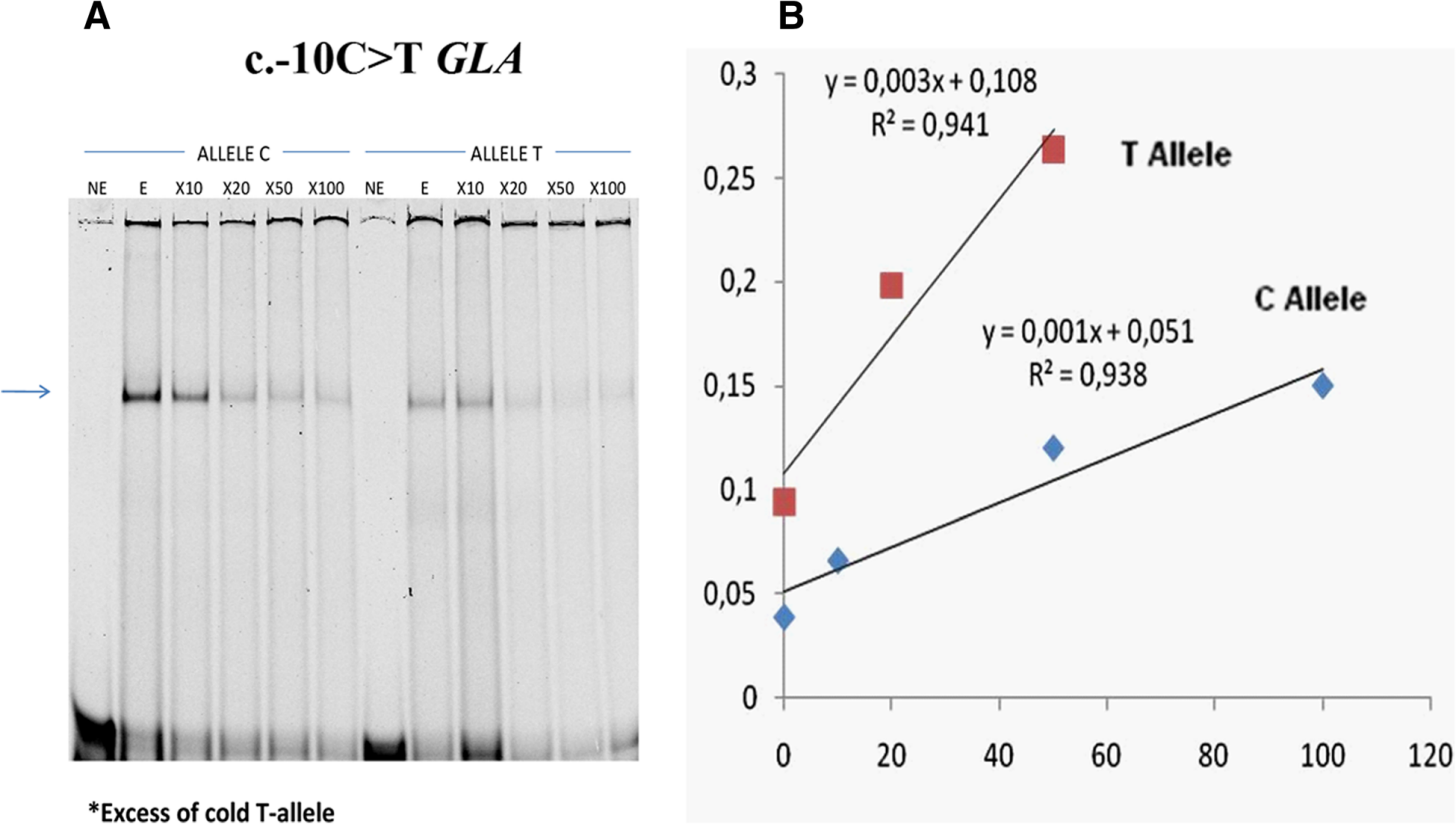
EMSA analysis. **a** EMSA carried out with probes containing the C or T allele for the IVSO-10C > T variant in the *GLA* gene promoter. **b** The inverse of band densities from the EMSA plotted against the excess of cold allele T oligonucleotides, showing that allele T (slope = 0.003) was more easily displaced from the complex than allele C (slope = 0.001)

To investigate whether the C > T change in the *GLA* gene promoter variant c.-10C > T impacted the binding capacity of possible transcription factors, EMSA was performed with specific primers for each variant. The results demonstrated marked differences in the affinity of nuclear proteins between the two alleles, with allele C having a greater ability to bind nuclear proteins (Fig. 6a). To confirm this difference, we performed a competitive binding EMSA using increasing amounts of an unlabelled oligonucleotide corresponding to the T allele. The results indicated that the T allele was more easily displaced from the protein-DNA complex (0.003 vs 0.001) (Fig. 6b). Therefore, the C > T substitution resulted in decreased protein binding capacity of the fragment. In an attempt to identify the transcription factors present in the complex, we scanned sequences surrounding the SNP c.-10C > T with MatInspector (Genomatix) [26]. The results of this analysis indicated that the mutation could affect the binding sites for small nuclear RNA (snRNA)-activating protein complex (SNAP-C), doublesex/mab-3 related (DMRT), and X box-binding factors.

### Splice-site score (SSS)

We analysed the variants of the CIH using SSPNN software. Changes in *GLA* splicing with CIH were observed only in c.370-81_370-77delCAGCC and c.640-16A > G variants. The c.370-81_370-77delCAGCC variant resulted in the disappearance of a possible acceptor site in IVS2-78, with a SSS of 0.80. The SSS for a normal acceptor site in intron 4 (IVS4-1) was raised from 0.58 to 0.62 with the c.640-16A > G variant. Although the SSS were minimal, the results indicated that the co-segregation of CIH variants may cause an abnormal splice pattern.

## Discussion

Detection of mutations in the *GLA* gene is essential to support clinical diagnosis of FD. Mutations in intronic regions can alter the *GLA* gene expression pattern in a manner related to different disease phenotypes and clinical manifestations [4, 5]. Intronic *GLA* variants often remain unidentified because these regions are not routinely evaluated by gene sequencing; consequently, the prevalence of FD may be underestimated [27, 28].

In the present study, we sequenced the entire *GLA* gene from genomic DNA of the family members and we used MLPA in an attempt to find alterations that could explain the FD-like characteristics observed. We identified a complex haplotype consisting of five intronic variants (c.-10C > T, c.369 + 990C > A, c.370-81_370-77delCAGCC, c.640-16A > G, c.1000-22C > T). In one study, 12 % of 740 subjects with clinically suspected FD showed polymorphisms in the *GLA* promoter region; of these, 99 % had simultaneous polymorphisms throughout the gene, and CIH formed by four of five variants observed in our study occurred in 9 % of these cases [11]. In a second study, Ferri and colleagues [13] identified five *GLA* haplotypes in non-coding regions in 67 female probands with FD manifestations. The most frequent of these was the CIH formed by four variants (13.4 %). Previous reports have found c.-10C > T, c.370-81_370-77delCAGCC, c.640-16A > G and c.1000-22C > T variants to be associated with different clinical manifestations including mild renal, neurological [10] and cardiac disorders [12]. It is important to know that depending on the sequencing design, these haplotypes might be the same type. We found FD-like symptoms (renal, cardiac and neurological involvement) associated with the family in our study (Table 1). In accordance with our results Apeland et al., described two unrelated families, one of them carrier of c.640-16A > G and c.1000-22C > T intronic variants, presenting a cardiomyopathy mimicking FD with normal enzymatic activity values and renal and cardiac deposits without accumulation of glycolipids in urine or plasma. In two patients, a 100-fold increase in Gb3 was observed in cardiac biopsies. Exon sequencing failed to detect heterozygosity in potential candidate genes [29].

We examined the c.-10C > T variant located in the *GLA* gene promoter region, which may be co-dominantly associated with a relatively decreased *GLA* expression at the level of transcription and/or translation [30]. By EMSA, we found that the T allele reduced the affinity of the nuclear protein binding site. A computer analysis using MatInspector showed that this region is a possible binding site for three families of transcription factors: SNAP-C, X box-binding factors (XBBF) and DMRT. SNAP-C binds to Oct-1 and TATA binding proteins (TBP), which are activators of snRNA and RNA polymerases, respectively [31]. X box-Protein 1 (XBP1) becomes initiated during the endoplasmic reticulum (ER) stress response [32]. In humans, the DMRT gene family encodes transcription factors that are related to the Drosophila double sex proteins [33]. Unfortunately, no tested antibodies are currently available to perform a supershift assay.

The c.-10 C > T variant is situated in a CpG island region (http://www.urogene.org/methprimer/). DNA methylation is a well-recognized epigenetic modifier in the control of gene expression. This reversible DNA modification takes place almost exclusively at cytosine residues that are associated with guanosine in CpG doublets, and mediates control of transcription through chromatin remodelling. This modification is widely implicated in various biological processes including X-inactivation, the regulation of tissue- and development-specific gene expression, foreign DNA inactivation and genomic imprinting [34]. FD symptoms exhibited by females carrying the T allele could partially depend on the methylation state of the C allele. Indeed, Bono and co-workers [11] reported a relationship between FD symptoms and polymorphisms in the promoter region. Future studies on the methylation states of the promoter region may provide more clues on these epigenetic effects in relation to phenotype.

We found low levels of wild-type transcript in some patients in agreement with previous reports [28, 35]. *GLA*-M transcript levels (3′ region of the intron 6 cloned fragment) were also altered with respect to their controls in most cases. The index case of this family (F1.1), a female heterozygous for CIH, presented significantly lower levels of wild-type transcript, whereas *GLA*-M expression was slightly reduced but not significantly different to control. Although she presented a cardiac phenotype, her leukocyte and plasma enzyme activities were not decreased. Higher residual activity is often found in atypical male patients who do not show the classic phenotype and have later onset of symptoms [36, 37], including cardiac [35] and renal variants [38]. It is possible that *GLA* expression in other tissues may be different. The ratio of the alternatively spliced transcript produced by another intronic variant, IVS4 + 919G > A mutation, to total α-Gal A mRNA, is higher in human muscle and lung tissues [27]. In the case of this studied family, the CIH only produced an altered *GLA* expression profile, presumably resulting in a late-onset FD-like phenotype. The differences observed in the qPCR assay may coincide with the SSS data variations for c.370-81_370-77delCAGCC and c.640-16A > G variants, the EMSA assay results for c.-10 C > T and also with the cDNA cloned fragment formed by a 49-bp segment of 3′ intron 6 and exon 7 in patient F1.4. However, PCR and sequencing did not reveal any products of splicing variants due to c.370-81_370-77delCAGCC or c.640-16A > G. It is possible that these transcripts are degraded by nonsense-mediated mRNA decay. In accord with the EMSA results, the expression levels of heterozygous CIH patients were approximately 50 % higher than in hemizygous carriers. Enzymatic activity was measured in plasma, lysed leukocytes and fibroblasts from CIH carriers and normal healthy controls. The mean value of lysed leukocyte activity in the CIH group was significantly lower (~20 %) but there was no significant difference between the plasma and fibroblast activity in control and CIH group. The expression is reduced in leukocytes reducing the enzyme activity but no the enzyme activity in other tissues like plasma supporting previous studies [30]. This effect may contribute to the glycolipid alteration and therefore may develop the clinical findings.

The levels of storage products in urine and plasma are elevated in most, but not all, FD patients. The demonstration of increased storage product levels is very useful in making a diagnosis in many cases and also for treatment monitoring. The predominant storage product in FD is Gb_3,_ but other storage products such as Ga_2_ or lyso- Gb_3_ may also accumulate. Consequently, significant differences in the Gb_3_ and Ga_2_ isoform profiles in urinary sediment were found amongst young, adult and atypical hemizygotes and heterozygotes using a combination of MALDI-TOF MS and tandem MS [39]. Additionally, increased sphingolipid storage in skin fibroblasts from patients has been described previously [40]. Therefore, we used an enzymatic fluorometric technique to quantify all galactosphingolipids in samples obtained from controls and CIH carriers in lysed leukocytes and skin fibroblasts. Galactose is a component of the headgroup of many glycolipids. Galactose oxidase specifically oxidizes the C-6 hydroxymethyl group of free galactose as well as all galactosyl derivatives, such as Gb_3_, lyso- Gb_3_ and Ga_2_, carrying a galactose residue in the terminal position. Bile acids, for example sodium taurocholate, do not alter the kinetics of galactose oxidase [41]. The enzymatic method to detect urinary Gb_3,_ showed a good recovery and comparability with a previously validated HPLC method [42]. Importantly, we validated this assay using a synthetic model of lysosomal storage in fibroblasts activated with TNFα and corroborated these findings with confocal microscopy quantification of CD77 (Gb3)/CD17(Lactosylceramide)/LAMP1. Confocal microscopy revealed that galactolipids were mostly confined to cytosolic and membrane compartments (Fig. 5b) in concordance with several studies that demonstrated that Gb3 in FD is not only present in lysosomes, but rather widely distributed in other cellular structures [43]. *In vitro* studies in skin fibroblasts showed that CIH carriers accumulated galactosphingolipids significantly after 5 days culture, in the range between 30 and 80 % in comparison with control samples. Fibroblasts from a male patient (F1.4) with a renal phenotype and glycolipid deposits demonstrated by renal biopsy, accumulated approximately 50 % more substrate compared with the F1.1 patient, a heterozygous female with a cardiac phenotype. In concordance with our results is important to remark that Namdar et al. demonstrated that vasculopathy in FD is directly caused by intracellular Gb3 accumulation while deficiency of *GLA* alone does not cause any deregulation of key vasoactive mediators [44].

Most of the carriers had high *in vitro* residual enzyme activity in plasma, leukocytes and cultured fibroblasts; however, CIH carriers had significant galactosphingolipid accumulation in fibroblasts in comparison with controls. Presumably, because the enzyme structure is not altered, only the *GLA* structural regulatory mechanism was affected by inheritance of the CIH, leading to *GLA* activation-dependent accumulation of substrates, influenced perhaps by environmental factors such as the proinflammatory state of the patient.

## Conclusions

CIH carriers showed a wide variation in residual enzymatic activities in leukocytes, plasma, and fibroblasts, but generally activity was normal. In contrast, galactosphingolipid accumulation was in the main significantly greater in fibroblasts compared with controls. Position −10 in the *GLA* promoter is a putative nuclear protein binding site situated in the CpG island region, acting as a gene regulatory zone. The inheritance of the co-segregated CIH variants alters the *GLA* expression pattern, producing a tissue glycolipid storage disorder.

The genetic analysis of the entire *GLA* gene sequence and MLPA, the study of *GLA* expression and glycolipid quantification in relation to FD clinical manifestations can be extremely helpful as tools for FD-related diagnosis.

Further studies are needed to elucidate how the inheritance of complex intronic haplotypes are implicated in the *GLA* regulatory mechanisms and therefore, the glycolipid metabolism alteration.

## Additional file

Additional file 1:
**Table S- Sequences of primers and probes.** (DOCX 21 kb)

## Abbreviations

*GLA*: Alfa galactosidase gene
FD: Fabry disease
Gb_3_: Globotriaosylcermide
Lyso-Gb_3_: Globotriaosylsphingosine
Ga_2_: Galabiosylceramide
α-Gal A: α-galactosidase A
CIH: Complex intronic haplotype
TNFα: Tumor necrosis factor alpha
LAMP1: Lysosomal-associated membrane protein 1
DGJ: 1-deoxygalactonojirimycin hydrochloride
MU: Methylumbelliferyl
PCR: Polymerase chain reaction
RT-PCR: Reverse transcription polymerase chain reaction
MLPA: Multiplex ligand probe amplification
FBS: Fetal bovine serum
DMEM: Dulbecco’s modified Eagle’s medium
qPCR: Quantitative real-time PCR
EMSA: Electrophoretic mobility shift assay
SSS: Splice-site score
MRI: Magnetic resonance image
LV: Left ventricle
LVEF: Left ventricular ejection fraction
MI: Mitral insufficiency
AI: Aortic insufficiency
QST: Quantitative sensory test
RQ: Relative quantification
Wt: Wild type
SNP: Single nucleotide polymorphism
snRNA: Small nuclear RNA
SNAP-C: Small nuclear RNA activating protein complex
XBBF: X box-binding factors
DMRT: Doublesex/mab-3 related
TBP: TATA binding proteins
