# Supplementary material for: Increased glycolipid storage produced by the inheritance of a complex intronic haplotype in the α-galactosidase A (GLA) gene
Source: BMC Genet. 2015 Sep 3;16:109. doi: 10.1186/s12863-015-0267-z (PMC4558912; doi:10.1186/s12863-015-0267-z)
Supplement: Additional file 1: — Table S- Sequences of primers and probes. (DOCX 21 kb) [file 12863_2015_267_MOESM1_ESM.docx]

## Table S- Sequences of primers and probes

| **Primer/probe** | **Sequence** |
| --- | --- |
| ***GLA* Sequencing** |  |
| IVS0_GLA_F | AACCCGTCCCATTTAATTCC |
| IVS0_GLA_R | CTCGCTATAGCCGTTTGAGG |
| Prom_GLA_F | CCACACACCAACCTCTAACGATAC |
| Prom_GLA_R | GGTGACCGGACAGCATAAAT |
| Exon1_GLA_F | TAGGGCGGGTCAATATCAAG |
| Exon1.1.0_GLA_R | TCACACAGAGAAAGTTTAGG |
| IVS1.a_GLA_F | CCCTTTGCTTTTCCATGTGT |
| IVS1.a_GLA_R | TTGCTCTGTAAGCGCAGTTG |
| IVS1.b_GLA_F | GGAAGGTTGACATGGAGGAA |
| IVS1.b_GLA_R | TTGCCATTAGCCAAACATGA |
| IVS1.c.2.0_GLA_F | CCAGAAATAATCATGGTTTAAATGTT |
| IVS1.c.2.0_GLA_R | GTATTATCTTCAGGTAGGAAAGGACTT |
| IVS1.d.1.0_GLA_F | AATACAGTGGTTCTTCCTATGTGTTGG |
| IVS1.d.1.0_GLA_R | GGTTTTGCATTTTGGCTAGGCTA |
| IVS1.e.1.0_GLA_F | TTTGTAATGTTGTGCCAAGTTCC |
| IVS1.e.1.0_GLA_R | ACCTCCCATTTATTAGGCACCTT |
| Exon2.1.0_GLA_F | GGAATATTAACGGGATAAGAGAGA |
| Exon2.1.0_GLA_R | ACTTTTAATAGAGTTGGGGTTTCA |
| IVS2.a.1.0_GLA_F | TACAGAAGCTTGTTTAGAAACAGC |
| IVS2.a.1.0_GLA_R | AAGGTTATATGGAAATACATGCAC |
| IVS2.b.1.0_GLA_F | ATGTTTTGTAATAGCTCTTGAGGC |
| IVS2.b.1.0_GLA_R | AATCTGCTCATTGGCTATAAATCT |
| IVS2.c_GLA_F | CTTGTAATCCGCCCACCTT |
| IVS2.c_GLA_R | CCCTAGCTTCAGTCCTTTGCT |
| Exon3’_GLA_F | CCCCAATACCTGGTGAAGTAA |
| Exon3’_GLA_R | TTTCCAGTATTGTGACAGGGTATTT |
| IVS3_GLA_F | CCAGAGATTTAGCCACAAAGG |
| IVS3_GLA_R | GAATTTCCAGCTGGGGCTAT |
| Exon4.1.0_GLA_F | GGAAGCTGAGACAGAAGAGT |
| Exon4.1.0_GLA_R | AGACACAAGGATGACTTTCC |
| IVS4.a_GLA_F | CCAGGTTCCAACCACTTCTC |
| IVS4.a_GLA_R | CCCTGCCCTCATGAAACTTA |
| IVS4.b_GLA_F | AGCCCTCTGTCCATTCATTCT |
| IVS4.b_GLA_R | TAATTGGGCTGTGAAAACAGA |
| Exon5.1.0_GLA_F | AAGGCTACAAGTGCCTCCTTT |
| IVS5_GLA_R | GGGCCATCTGAGTTACTTGC |
| Exon6_GLA_F | CTCCTTGTTCAAGACCCTGCGGTAG |
| IVS6_GLA_R | GGGAAGCAACTGCGATGGTATAAGA |
| Exon7_GLA_F | CCAAACTAACAGGGCCACTT |
| Exon7_GLA_R | ATGAGCCACCTAGCCTTGAG |
| **mRNA *GLA* amplificatio**n |  |
| GLA20F | TGC AGC TGA GGA ACC CAG AAC TACA |
| GLA613R | AGA GGC CAC TCA CAG GAG TAC AC |
| GLA497F | CTG ACT GGG GAG TAG ATC TGC TAA |
| GLA1113R | AGG GAA GCA ACT GCG ATG GTA |
| **qPCR** |  |
| GLA wt* | Hs00609238_M1 Ref.4331182 |
| GLA 2F | TGCCAAACTAACAGGGCCAC |
| GLA 2R | CCTGCCGGTTTATCATAGCTAC |
| GLA2 probe | AAG CCTGAGAGAGGTCGTTCCC |
| GAPDH* | Ref.4331182 |
| **RNA Cloning fragment** |  |
| I6** | MSC0000934 |
| miScript Universal Primer** | Varies |
| T7F | TAATACGACTCACTATAGGG |
| Sp6R | GATTTAGGTGACACTATAG |
| **EMSA** | GCTGTCCGGT[C/T]ACCGTGACAA |

F=forward, R=reverse, Source:* Applied Biosystems, ** Qiagen.
